# Supplementary material for: Inflammation and Insulin Resistance in Diabetic Chronic Coronary Syndrome Patients
Source: Nutrients. 2023 Jun 19;15(12):2808. doi: 10.3390/nu15122808 (PMC10301506; doi:10.3390/nu15122808)
Supplement: Supplementary file 1 [file nutrients-15-02808-s001.zip › nutrients-2402543-supplementary.pdf]

Supplementary materials for

Inflammation and Insulin Resistance in Diabetic Chronic Coronary Syndrome Patients

Tianyu Li, Peizhi Wang, Xiaozeng Wang, Zhenyu Liu, Zheng Zhang, Yongzhen Zhang, Zhifang Wang, Yingqing Feng, Qingsheng Wang, Xiaogang Guo, Xiaofang Tang, Jingjing Xu, Ying Song, Yan Chen, Na Xu, Yi Yao, Ru Liu, Pei Zhu, Yaling Han and Jinqing Yuan

Table S1. Number of missing values and corresponding dispositions.
Table S2. Univariable Cox proportional-hazard regression analysis for clinical outcomes.
Table S3. Association of TyG or hsCRP with cardiovascular events.
Table S4. Sensitivity analyses of the associations of TyG and hsCRP with MACE.
Figure S1. Kaplan-Meier analysis of patients grouped by TyG levels.
Figure S2. Kaplan-Meier analysis of patients grouped by TyG and hsCRP levels.
Figure S3. Restricted cubic spline curves of TyG or hsCRP for cardiovascular events.
Figure S4. Spearman correlation between TyG and hsCRP.

Table S1. Number of missing values and corresponding dispositions.

	Number of missing values	Disposition
Body mass index	8 (0.2%)	Median imputation
Glycated hemoglobin	44 (1.0%)	Median imputation
Left ventricular ejection fraction	38 (0.9%)	Median imputation
SYNTAX score	2 (0.0%)	Undisposed
Aspirin adherence	57 (1.3%)	Mode imputation
Statins adherence	53 (1.2%)	Mode imputation

Abbreviations: SYNTAX, synergy between percutaneous coronary intervention with Taxus and cardiac surgery

Table S2. Univariable Cox proportional-hazard regression analysis for clinical outcomes.

Variables	MACE		All-cause death		Cardiac death		Myocardial infarction		Any revascularization	
	**HR (95% CI)**	***p***	**HR (95% CI)**	***p***	**HR (95% CI)**	***p***	**HR (95% CI)**	***p***	**HR (95% CI)**	***p***
Age, years	1.00 (0.99, 1.01)	0.605	1.07 (1.04, 1.09)	<0.001	1.07 (1.04, 1.10)	<0.001	1.01 (0.99, 1.03)	0.234	0.99 (0.97, 1.00)	0.033
≥65	1.00 (0.81, 1.22)	0.973	2.67 (1.73, 4.14)	<0.001	2.44 (1.40, 4.26)	0.002	1.01 (0.69, 1.46)	0.967	0.83 (0.65, 1.06)	0.142
Female	0.81 (0.64, 1.01)	0.075	0.85 (0.52, 1.39)	0.510	1.30 (0.72, 2.32)	0.382	0.76 (0.50, 1.17)	0.220	0.74 (0.56, 0.97)	0.032
BMI, kg/m^2	1,02 (0.99, 1.05)	0.251	0.97 (0.91, 1.04)	0.440	0.96 (0.88 1.05)	0.427	1.02 (0.96, 1.07)	0.545	1.03 (0.99, 1.06)	0.119
≥28	1.19 (0.97, 1.47)	0.099	0.92 (0.57, 1.50)	0.739	0.82 (0.43, 1.56)	0.536	1.03 (0.69, 1.54)	0.889	1.30 (1.01, 1.66)	0.041
Smoking history										
Current smoker	1.27 (1.02, 1.57)	0.030	1.02 (0.64, 1.60)	0.947	1.07 (0.59, 1.94)	0.830	1.59 (0.98, 2.59)	0.062	1.33 (0.95, 1.85)	0.097
Former smoker	1.22 (0.92, 1.61)	0.164	0.73 (0.37, 1.42)	0.354	0.96 (0.43, 2.17)	0.930	1.29 (0.86, 1.54)	0.889	1.43 (1.10, 1.86)	0.007
Non-Smoker	Reference		Reference		Reference		Reference		Reference	
Insulin use	1.72 (1.36, 2.17)	<0.001	1.58 (0.94, 2.66)	0.084	1.64 (0.84, 3.20)	0.145	1.60 (1.03, 2.49)	0.037	1.83 (1.39, 2.41)	<0.001
Hypertension	1.04 (0.70, 1.56)	0.835	1.41 (0.52, 3.85)	0.502	3.44 (0.48, 24.94)	0.221	1.30 (0.57, 2.95)	0.532	0.84 (0.54, 1.32)	0.456
Dyslipidemia	0.96 (0.56, 1.63)	0.877	0.55 (0.22, 1.35)	0.190	0.40 (0.15, 1.12)	0.081	1.32 (0.42, 4.16)	0.634	1.35 (0.64, 2.86)	0.432
PAD	1.51 (1.10, 2.06)	0.010	1.07 (0.50, 2.33)	0.855	1.61 (0.69, 3.78)	0.271	1.48 (0.83, 2.63)	0.183	1.71 (1.19, 2.45)	0.003
CKD	1.68 (1.10, 2.56)	0.016	5.04 (2.79, 9.09)	<0.001	5.15 (2.42, 10.96)	<0.001	2.58 (1.35, 4.93)	0.004	1.12 (0.62, 2.06)	0.703
COPD	1.08 (0.48, 2.43)	0.844	0.85 (0.12, .6.07)	0.867	1.43 (0.20, 10.38)	0.721	1.84 (0.59, 5.80)	0.296	0.76 (0.24, 2.38)	0.639
Prior MI	1.40 (1.13, 1.74)	0.002	2.67 (1.73, 4.10)	<0.001	3.83 (2.21, 6.64)	<0.001	1.79 (1.22, 2.62)	0.003	1.24 (0.95, 1.63)	0.110
Prior stroke	1.69 (1.35, 2.11)	<0.001	1.25 (0.74, 2.10)	0.406	1.13 (0.57, 2.26)	0.729	1.29 (0.90, 1.87)	0.168	1.62 (1.24, 2.12)	<0.001
Prior revascularization	1.29 (1.06, 1.57)	0.012	1.86 (1.22, 2.85)	0.004	2.13 (1.23, 3.70)	0.007	1.74 (1.16, 2.61)	0.008	1.33 (1.05, 1.69)	0.018
FBG, mmol/L	1.03 (0.99, 1.06)	0.132	1.07 (0.99, 1.14)	0.007	1.05 (0.95, 1.15)	0.325	0.98 (0.91, 1.05)	0.576	1.04 (1.00, 1.09)	0.053
HbA1c, %	1.09 (1.02, 1.17)	0.018	1.20 (1.04, 1.39)	0.013	1.21 (1.00, 1.45)	0.048	1.04 (0.91, 1.19)	0.556	1.09 (1.00, 1.19)	0.043
LDL-c, mmol/L	1.09 (0.97, 1.21)	0.133	1.13 (0.89, 1.43)	0.321	1.26 (0.94, 1.68)	0.122	0.96 (0.78, 1.19)	0.720	1.13 (0.99, 1.28)	0.070
>1.8	1.11 (0.89, 1.38)	0.367	1.12 (0.70, 1.81)	0.636	1.10 (0.60, 2.04)	0.760	0.95 (0.64, 1.40)	0.783	1.24 (0.95, 1.63)	0.114
Triglyceride, mmol/L	1.07 (1.00, 1.14)	0.060	0.88 (0.69, 1.13)	0.317	0.97 (0.75, 1.26)	0.826	1.07 (0.95, 1.21)	0.293	1.08 (1.01, 1.16)	0.031
LVEF <40%	2.06 (1.25, 3.40)	0.005	7.70 (4.08, 14.52)	<0.001	10.88 (5.30, 22.37)	<0.001	2.61 (1.15, 5.93)	0.022	0.92 (0.38, 2.23)	0.855
LM/TVD	1.71 (1.40, 2.10)	<0.001	2.72 (1.68, 4.39)	<0.001	3.30 (1.73, 6.29)	<0.001	2.06 (1.40, 3.02)	<0.001	1.49 (1.17, 1.88)	0.001
SYNTAX score										
≤22	Reference		Reference		Reference		Reference		Reference	
23-32	1.53 (1.18, 1.99)	0.001	1.99 (1.16, 3.42)	0.013	2.41 (1.21, 4.79)	0.012	1.40 (0.85, 2.30)	0.181	1.45 (1.06, 1.99)	0.020
≥33	2.55 (1.8, 3.86)	<0.001	5.35 (2.73, 10.49)	<0.001	9.31 (4.43, 19.56)	<0.001	2.38 (1.10, 5.14)	0.027	1.78 (1.00, 3.19)	0.051

PCI status										
Successful PCI	Reference		Reference		Reference		Reference		Reference	
Unsuccessful PCI	2.54 (1.72, 3.76)	<0.001	2.89 (1.02, 8.19)	0.047	3.90 (0.86, 17.60)	0.077	1.64 (0.71, 3.76)	0.243	2.93 (1.90, 4.53)	<0.001
No PCI	0.92 (0.74, 1.14)	0.431	3.09 (1.97, 4.85)	<0.001	6.12 (3.13, 11.97)	<0.001	0.77 (0.51, 1.15)	0.196	0.79 (0.61, 1.03)	0.081
Aspirin adherence										
2-year regular	0.33 (0.24, 0.44)	<0.001	0.01 (0.00, 0.02)	<0.001	0.01 (0.01, 0.03)	<0.001	0.38 (0.21, 0.68)	0.001	0.68 (0.39, 1.17)	0.161
1-year regular	0.40 (0.28, 0.56)	<0.001	0.13 (0.08, 0.21)	<0.001	0.14 (0.08, 0.26)	<0.001	0.48 (0.25, 0.92)	0.027	0.73 (0.44, 1.19)	0.208
Irregular/<1 year	Reference		Reference		Reference		Reference		Reference	
Statins adherence										
2-year regular	1.24 (0.89, 1.74)	0.205	1.79 (0.77, 4.16)	0.173	2.18 (0.67, 7.09)	0.1950	0.99 (0.51, 1.90)	0.970	1.38 (0.89, 2.13)	0.147
1-year regular	1.28 (0.89, 1.84)	0.184	1.63 (0.66, 4.02)	0.289	1.91 (0.54, 6.70)	0.3132	1.10 (0.61, 1.98)	0.755	1.23 (0.82, 1.84)	0.320
Irregular/<1 year	Reference		Reference		Reference		Reference		Reference	

Abbreviations: BMI, body mass index; CAD, coronary artery disease; CKD, chronic kidney disease; COPD, chronic obstructive pulmonary disease; FBG, fasting blood glucose; HbA1c, glycated hemoglobin; hsCRP, high-sensitivity C-reactive protein; LDL-c, low-density lipoprotein cholesterol; LM/TVD, left main stem/three-vessel disease; LVEF, left ventricular ejection fraction; MACE, major adverse cardiac event; MI, myocardial infarction; PAD, peripheral artery disease; PCI, percutaneous coronary intervention; SYNTAX, synergy between percutaneous coronary intervention with Taxus and cardiac surgery; TyG, triglyceride-glucose index.

Table S3. Association of TyG or hsCRP with cardiovascular events.

**Clinical outcome**	**Group**	**No. of events (%)**	**Event rate per 1000 py**	**Crude model HR (95% CI)**	***p***	**Adjusted model HR (95% CI)**	***p***
MACE	TyG (per SD)			1.23 (1.05, 1.43)	0.008	1.18 (1.06, 1.33)	0.033
	<8.46	50 (6.19)	29.49	Reference		Reference	
	≥8.46	355 (9.83)	47.12	1.60 (1.19, 2.14)	0.002	1.59 (1.18, 2.15)	0.003
	hsCRP (per SD)			1.03 (1.01, 1.04)	0.007	1.02 (1.00, 1.04)	0.041
	<2.00 mg/L	235 (8.53)	40.90	Reference		Reference	
	≥2.00 mg/L	170 (10.22)	48.80	1.19 (1.02, 1.63)	0.031	1.10 (0.99, 1.34)	0.060
	p for interaction[a]			0.216			
All-cause death	TyG (per SD)			1.28 (0.98, 1.54)	0.058	1.77 (1.18, 2.66)	0.005
	<8.46	9 (1.11)	5.13	Reference		Reference	
	≥8.46	76 (2.10)	9.59	1.86 (0.93, 3.72)	0.078	2.91 (1.41, 6.01)	0.004
	hsCRP (per SD)			1.05 (1.03, 1.07)	<0.001	1.03 (1.00, 1.07)	0.054
	<2.00 mg/L	42 (1.52)	6.98	Reference		Reference	
	≥2.00 mg/L	43 (2.58)	11.74	1.68 (1.10, 2.57)	0.017	1.43 (1.02, 2.21)	0.047
	p for interaction[a]			0.392			
Cardiac death	TyG (per SD)			1.36 (1.01, 1.79)	0.048	1.96 (1.23, 3.12)	0.005
	<8.46	5 (0.62)	2.85	Reference		Reference	
	≥8.46	46 (1.27)	5.81	2.07 (0.82, 5.20)	0.123	3.04 (1.15, 8.02)	0.024
	hsCRP (per SD)			1.06 (1.03, 1.08)	<0.001	1.05 (1.01, 1.09)	0.007
	<2.00 mg/L	24 (0.87)	3.99	Reference		Reference	
	≥2.00 mg/L	27 (1.62)	7.37	1.86 (1.07, 3.23)	0.027	1.52 (1.03, 2.69)	0.047
	p for interaction[a]			0.204			
Myocardial infarction	TyG (per SD)			1.14 (0.86, 1.51)	0.376	1.05 (0.66, 1.66)	0.845
	<8.46	10 (1.24)	5.75	Reference		Reference	
	≥8.46	108 (2.99)	13.87	2.41 (1.26, 4.61)	0.008	2.42 (1.25, 4.67)	0.009
	hsCRP (per SD)			1.04 (1.02, 1.06)	<0.001	1.04 (1.01, 1.06)	0.005
	<2.00 mg/L	61 (2.21)	10.29	Reference		Reference	
	≥2.00 mg/L	57 (3.43)	15.83	1.54 (1.07, 2.20)	0.020	1.45 (1.01, 2.09)	0.045
	p for interaction[a]			0.290			
Any revascularization	TyG (per SD)			1.30 (1.09, 1.56)	0.004	1.20 (1.03, 1.56)	0.026
	<8.46	38 (4.70)	22.26	Reference		Reference	
	≥8.46	244 (6.76)	31.94	1.35 (1.06, 1.72)	0.015	1.33 (1.04, 1.88)	0.028
	hsCRP (per SD)			1.01 (0.98, 1.04)	0.581	1.01 (0.98, 1.04)	0.705

	<2.00 mg/L	167 (6.06)	28.72	Reference		Reference	
	≥2.00 mg/L	115 (6.91)	32.56	1.13 (0.89, 1.43)	0.326	1.07 (0.84, 1.36)	0.605
	p for interaction[a]			0.914			

[a] Interaction between TyG category and hsCRP category.

Adjusted for age (continuous), sex, body mass index (continuous), smoking history, insulin use, peripheral artery disease, left ventricular ejection fraction <40%, left main stem/three-vessel disease, synergy between percutaneous coronary intervention with Taxus and cardiac surgery score (categorical), percutaneous coronary intervention status, aspirin adherence, statins adherence. TyG was additionally adjusted for hsCRP, and hsCRP was additionally adjusted for TyG. Abbreviations: CI, confidence interval; H, high; HR, hazard ratio; hsCRP, high-sensitivity C-reactive protein; L, low; MACE, major adverse cardiac event; No., number; py, person-year; SD, standard deviation; TyG, triglyceride-glucose index.

Table S4. Sensitivity analyses of the associations of TyG and hsCRP with MACE.

Group	No. of events (%)	Event rate per 1000 pys	Crude model HR (95% CI)	Crude model *p*	Adjusted model HR (95% CI)	Adjusted model *p*
**Combining TyG and hsCRP into 9 groups [a]**						
L-TyG/L-hsCRP	20 (5.09)	24.25	Reference		Reference	
L-TyG/M-hsCRP	19 (7.09)	33.26	1.50 (0.81, 2.76)	0.350	1.30 (0.70, 2.41)	0.413
L-TyG/H-hsCRP	11 (8.80)	43.34	1.75 (0.84, 3.64)	0.138	1.54 (0.75, 3.16)	0.239
M-TyG/L-hsCRP	68 (7.86)	38.19	1.57 (0.95, 2.59)	0.076	1.38 (0.86, 2.16)	0.072
M-TyG/M-hsCRP	87 (9.54)	44.70	1.87 (1.08, 3.64)	0.026	1.73 (1.06, 2.84)	0.029
M-TyG/H-hsCRP	65 (11.07)	53.25	2.06 (1.14, 3.73)	0.017	1.88 (1.04, 3.44)	0.035
H-TyG/L-hsCRP	25 (8.36)	41.18	1.74 (1.06, 2.87)	0.030	1.51 (1.01, 2.46)	0.041
H-TyG/M-hsCRP	67 (11.07)	52.89	2.06 (1.28, 3.30)	0.003	1.88 (1.12, 3.15)	0.016
H-TyG/H-hsCRP	43 (11.78)	56.70	2.17 (1.27, 3.71)	0.005	2.07 (1.24, 3.44)	0.005
**Excluding patients who did not complete 2-year follow-up (N=4362)**						
L-TyG/L-hsCRP	32 (5.40)	25.33	Reference		Reference	
L-TyG/H-hsCRP	18 (8.87)	42.95	1.69 (0.95, 3.01)	0.075	1.46 (0.82, 2.61)	0.200
H-TyG/L-hsCRP	203 (9.55)	45.69	1.80 (1.24, 2.61)	0.002	1.78 (1.22, 2.59)	0.003
H-TyG/H-hsCRP	152 (10.56)	49.95	1.96 (1.34, 2.87)	0.001	1.82 (1.24, 2.70)	0.003
**Excluding patients with hsCRP >10 mg/L (N=4190)**						
L-TyG/L-hsCRP	32 (5.32)	25.11	Reference		Reference	
L-TyG/H-hsCRP	13 (7.56)	36.51	1.45 (0.76, 2.76)	0.261	1.34 (0.70, 2.57)	0.372
H-TyG/L-hsCRP	203 (9.43)	45.40	1.81 (1.25, 2.62)	0.002	1.78 (1.22, 2.61)	0.003
H-TyG/H-hsCRP	128 (10.13)	47.96	1.91 (1.30, 2.81)	0.001	1.78 (1.20, 2.65)	0.004
**Excluding patients with imputed values (N=4222)**						
L-TyG/L-hsCRP	32 (5.50)	26.07	Reference		Reference	
L-TyG/H-hsCRP	17 (8.67)	41.93	1.60 (0.89, 2.89)	0.116	1.40 (0.78, 2.54)	0.261
H-TyG/L-hsCRP	198 (9.61)	46.09	1.76 (1.21, 2.56)	0.003	1.74 (1.19, 2.53)	0.004
H-TyG/H-hsCRP	148 (10.66)	50.49	1.92 (1.31, 2.82)	0.001	1.76 (1.19, 2.61)	0.005

[a] TyG was categorized at 8.46 and 9.31 and hsCRP was categorized at 1.00 and 3.00.

Adjusted for age (continuous), sex, body mass index (continuous), smoking history, insulin use, peripheral artery disease, left ventricular

ejection fraction <40%, left main stem/three-vessel disease, synergy between percutaneous coronary intervention with Taxus and cardiac surgery score (categorical), percutaneous coronary intervention status, aspirin adherence, statins adherence. Abbreviations: CI, confidence interval; H, high; HR, hazard ratio; hsCRP, high-sensitivity C-reactive protein; L, low; M-, moderate; MACE, major adverse cardiac event; No., number; py, person-year; TyG, triglyceride-glucose index.

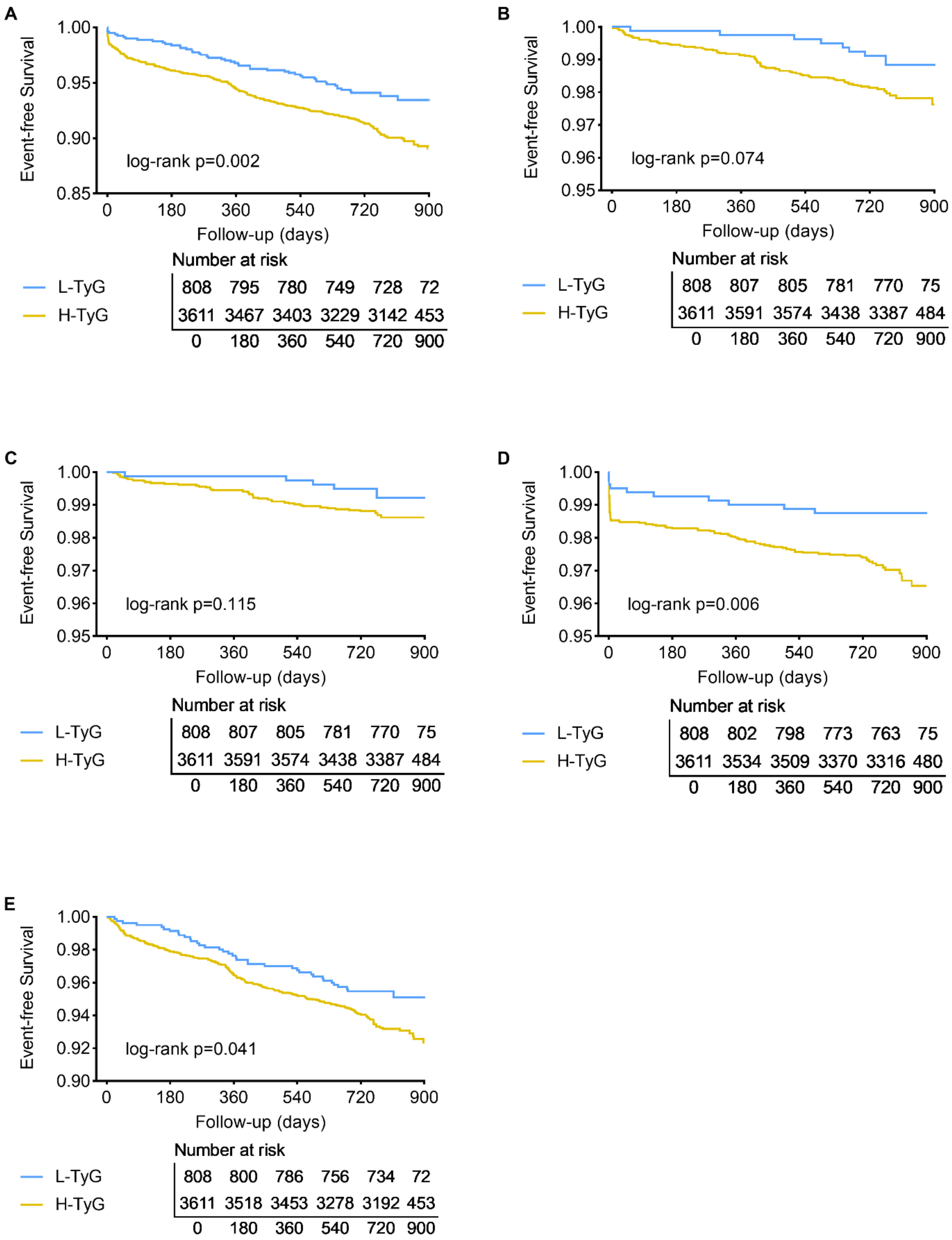

Figure S1. Kaplan-Meier analysis of patients grouped by TyG levels.
Survival curves for MACE (A), all-cause death (B), cardiac death (C), myocardial infarction (D), and any revascularization (E). Abbreviations: H, high; L, low; MACE, major adverse cardiac events; TyG, triglyceride-glucose index.

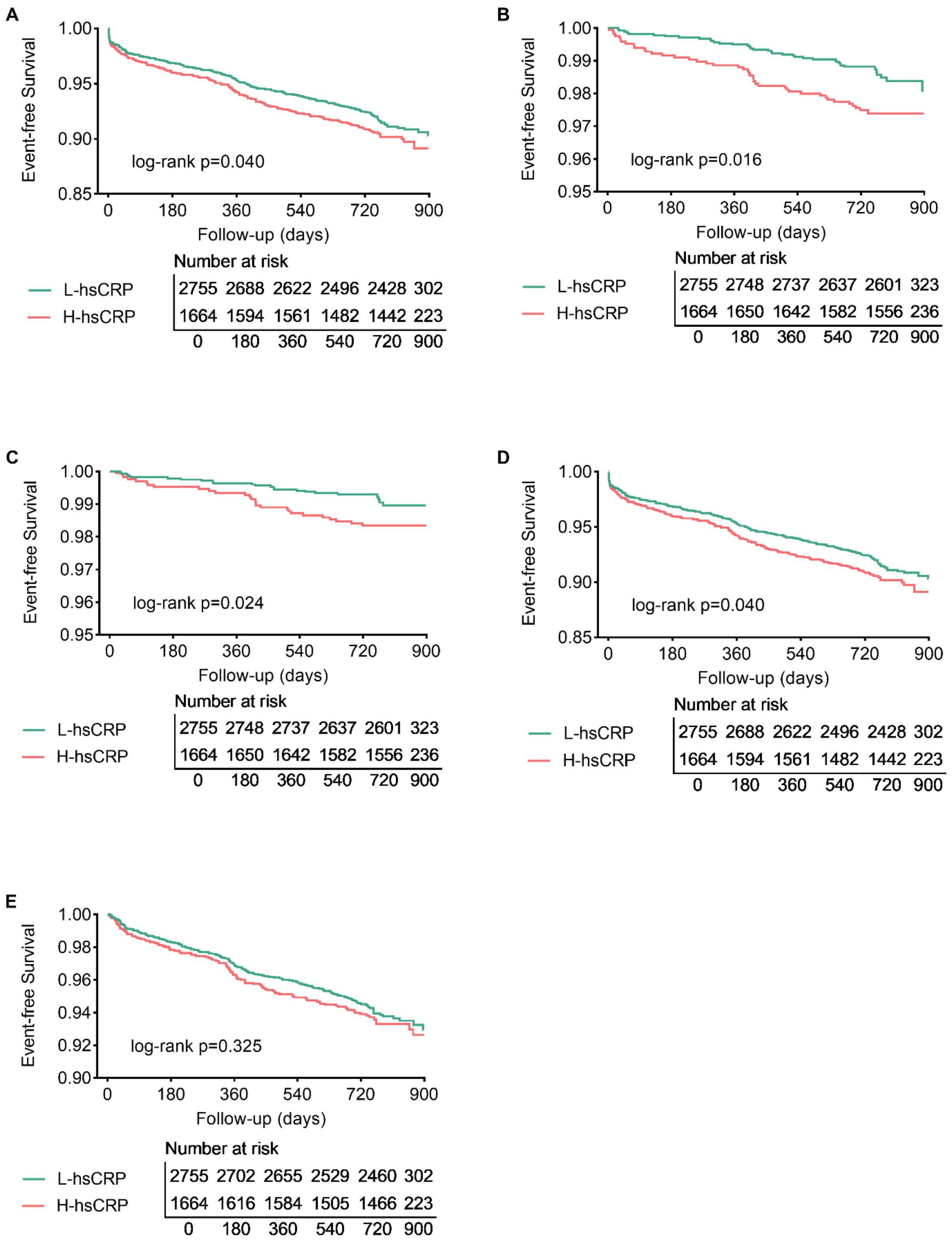

Figure S2. Kaplan-Meier analysis of patients grouped by TyG and hsCRP levels. Survival curves for MACE (A), all-cause death (B), cardiac death (C), myocardial infarction (D), and any revascularization (E). Abbreviations: H, high; hsCRP, high-sensitivity C-reactive protein; L, low; MACE, major adverse cardiac events.

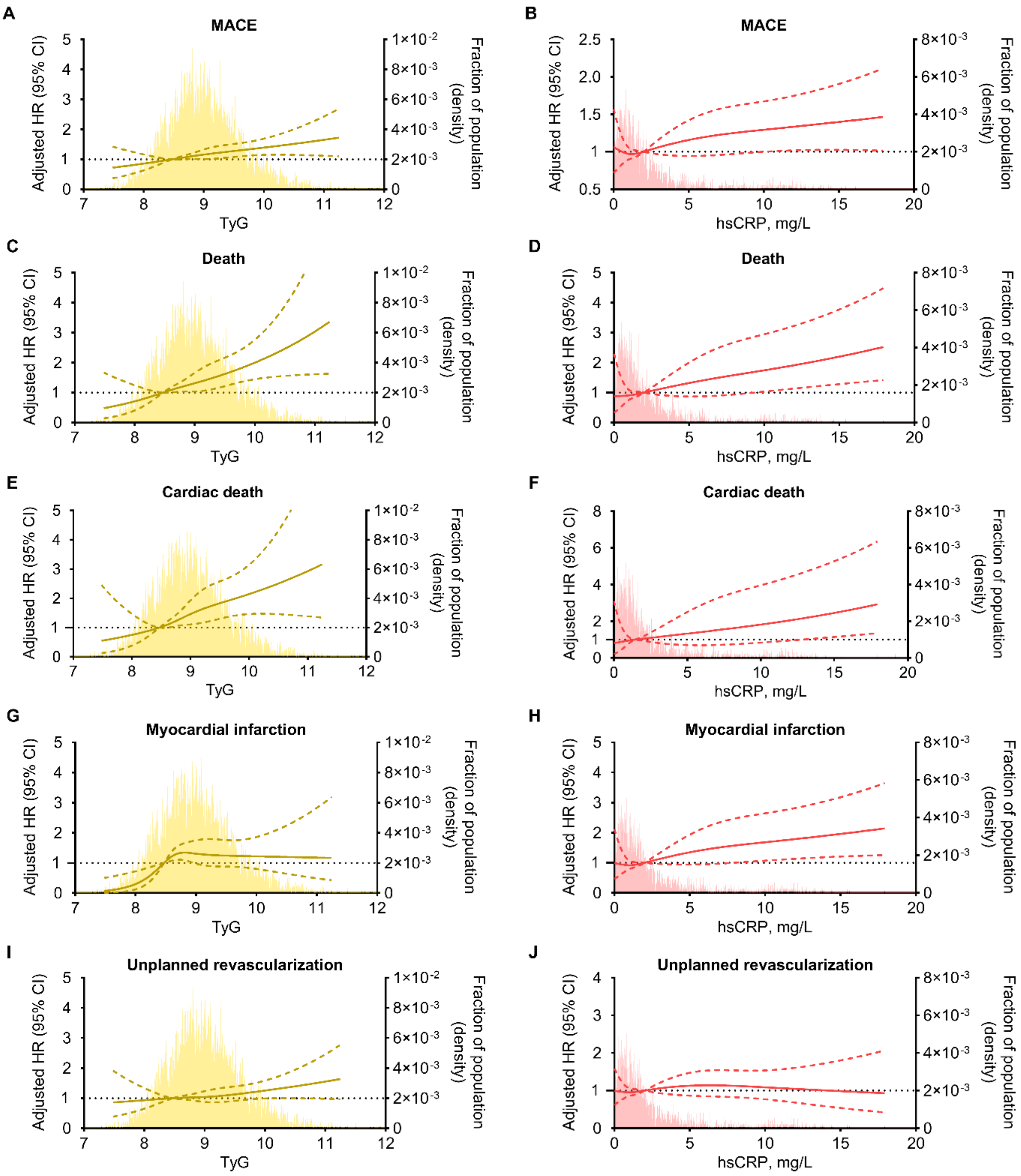

Figure S3. Restricted cubic spline curves of TyG or hsCRP for cardiovascular events. A TyG level of 8.46 and an hsCRP level of 2.00 mgl/L were set as the reference. Adjusted for age (continuous), sex, BMI (continuous), smoking history, peripheral art”
ery disease, chronic kidney disease, prior myocardial infarction, prior stroke, prior revascularization, low-density lipoprotein cholesterol ≤1.8 mmol/L; left ventricular ejection fraction <40%, left main stem/three-vessel disease, synergy between percutaneous coronary intervention with Taxus and cardiac surgery score (categorical), percutaneous coronary intervention status, aspirin adherence, statins adherence. Abbreviations: CI, confidence interval; HR, hazard ratio; hsCRP, high-sensitivity C-reactive protein; MACE, major adverse cardiac event; TyG, triglyceride-glucose index.

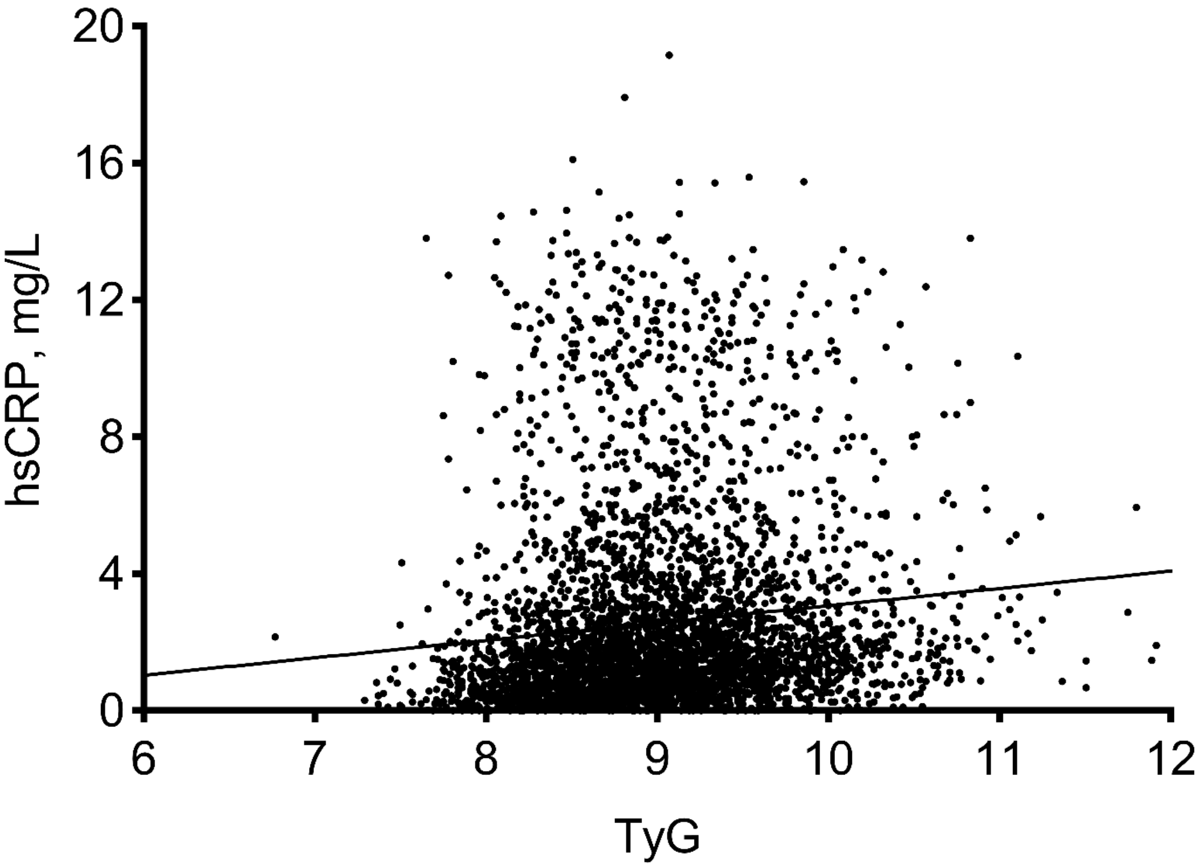

Spearman correlation r = 0.196 *p*<0.001

Figure S4. Spearman correlation between TyG and hsCRP.
Abbreviations: hsCRP, high-sensitivity C-reactive protein; TyG, triglyceride-glucose index.
